# Characterization and Implications of IncP-2A Plasmid pMAS152 Harboring Multidrug Resistance Genes in Extensively Drug-Resistant *Pseudomonas aeruginosa*

**DOI:** 10.3390/microorganisms12030562

**Published:** 2024-03-12

**Authors:** Li Mei, Yang Song, Xiao Liu, Kun Li, Xu Guo, Li Liu, Yang Liu, Zisis Kozlakidis, Io Hong Cheong, Duochun Wang, Qiang Wei

**Affiliations:** 1 National Pathogen Resource Center, Chinese Center for Disease Control and Prevention, Beijing 102206, China; ml980821@163.com (L.M.); liuli12342022@163.com (L.L.); lyioiyl@163.com (Y.L.); 2 Division of Infectious Disease, National Key Laboratory of Intelligent Tracking and Forecasting for Infectious Diseases, Chinese Center for Disease Control and Prevention, Beijing 102206, China; songyang@chinacdc.cn; 3 National Key Laboratory of Intelligent Tracking and Forecasting for Infectious Diseases (NITFID), National Institute for Communicable Disease Control and Prevention, Chinese Center for Disease Control and Prevention, Beijing 102206, China; liuxiao930919@163.com (X.L.); likun0310@hotmail.com (K.L.); 4 National Immunization Program, Chinese Center for Disease Control and Prevention, Beijing 100050, China; guoxu@chinacdc.cn; 5 International Agency for Research on Cancer, World Health Organization, 69007 Lyon, France; kozlakidisz@iarc.who.int; 6 State Key Laboratory of Systems Medicine for Cancer, Centre for Single-Cell Omics, School of Public Health, Shanghai Jiao Tong University School of Medicine, Shanghai 200127, China; cheong.iohong@shsmu.edu.cn

**Keywords:** extensively drug-resistant (XDR), *Pseudomonas aeruginosa*, plasmid, antimicrobial resistance genes (ARGs), *rmtB*, *bla*_PER_

## Abstract

Bacterial antimicrobial resistance (AMR) poses a significant global public health challenge. The escalation of AMR is primarily attributed to the horizontal gene transfer (HGT) of antibiotic resistance genes (ARGs), often facilitated by plasmids. This underscores the critical need for a comprehensive understanding of the resistance mechanisms and transmission dynamics of these plasmids. In this study, we utilized in vitro drug sensitivity testing, conjugation transfer assays, and whole-genome sequencing to investigate the resistance mechanism of an extensively drug-resistant (XDR) *Pseudomonas aeruginosa* clinical isolate, MAS152. We specifically focused on analyzing the drug-resistant plasmid pMAS152 it harbors and its potential for widespread dissemination. Bioinformatics analysis revealed that MAS152 carries a distinct IncpP-2A plasmid, pMAS152, characterized by a 44.8 kb multidrug resistance (MDR) region. This region houses a 16S rRNA methyltransferase (16S-RMTase) gene, *rmtB*, conferring high-level resistance to aminoglycoside antibiotics. Notably, this region also contains an extended-spectrum β-Lactamase (ESBL) gene, *bla*_PER-1_, and an efflux pump operon, *tmexCD-oprJ*, which mediate resistance to β-Lactams and quinolone antibiotics, respectively. Such a combination of ARGs, unprecedented in reported plasmids, could significantly undermine the effectiveness of first-line antibiotics in treating *P. aeruginosa* infections. Investigation into the genetic environment of the MDR region suggests that *Tn*2 and *IS*91 elements may be instrumental in the horizontal transfer of *rmtB*. Additionally, a complex Class I integron with an *IS*CR1 structure, along with *Tn*As1, seems to facilitate the horizontal transfer of *bla*_PER-1_. The conjugation transfer assay, coupled with the annotation of conjugation-related genes and phylogenetic analysis, indicates that the plasmid pMAS152 functions as a conjugative plasmid, with other genus *Pseudomonas* species as potential hosts. Our findings provide vital insights into the resistance mechanisms and transmission potential of the XDR *P. aeruginosa* isolate MAS152, underlining the urgent need for novel strategies to combat the spread of AMR. This study highlights the complex interplay of genetic elements contributing to antibiotic resistance and underscores the importance of continuous surveillance of emerging ARGs in clinical isolates.

## 1. Introduction

The threat posed by bacterial antimicrobial resistance (AMR) is escalating globally. The World Health Organization (WHO) highlighted “combatting drug resistance” as a primary global health concern in 2021 [1]. Alarmingly, AMR-related infections led to approximately 1.27 million deaths worldwide in 2019 [2], with projections estimating over 10 million annual deaths by 2050 [3]. This crisis is expected to incur substantial economic burdens, with an anticipated USD 1 trillion increase in global health expenditures annually by 2050, potentially exacerbating poverty levels [4].

“ESKAPE” pathogens, encompassing *Enterococcus faecium* (E), *Staphylococcus aureus* (S), *Klebsiella pneumoniae* (K), *Acinetobacter baumannii* (A), *Pseudomonas aeruginosa* (P), and *Enterococci* (E), are notorious for their easy acquisition of antibiotic resistance and high mortality rates [5]. Among them, *P. aeruginosa*, a prevalent cause of nosocomial infections, poses significant challenges mainly due to its high level of resistance to several antibiotics. In 2018, the WHO listed carbapenem-resistant *P. aeruginosa* (CRPA) as one of the “critical”-priority resistant bacteria in the development of new antibiotics [6]. *P. aeruginosa* has natural resistance mechanisms to many antibiotics, including low outer membrane permeability and efflux pump expression [7,8]. Current treatments for *P. aeruginosa* infections are increasingly compromised by AMR, attributed largely to the horizontal gene transfer (HGT) of ARGs, often mediated by plasmids [9,10]. Drug-resistant plasmids typically harbor an accessory module carrying diverse ARGs, endowing the host with antibiotic resistance. Furthermore, plasmid conjugation can lead to the prevalence of ARGs in different host strains, plasmid rearrangements, and evolution [11]. Incidents like the *bla*_IMP-45_-bearing IncP-2 plasmid-induced CRPA outbreak in Shanghai and the global spread of *mcr* genes underline the urgency of addressing plasmid-mediated resistance [12,13,14].

Although plasmids play an important driving role in the dissemination of AMR in *P. aeruginosa*, little is currently known about the potential evolutionary mechanism of resistance plasmids. This study presents the complete nucleotide sequence of the IncP-2 plasmid pMAS152 from an extensively drug-resistant (XDR) *P. aeruginosa* clinical isolate, MAS152. The plasmid carries significant ARGs including *rmtB*, *bla*_PER-1,_ and *tmexCD-oprJ.* We aim to elucidate its genetic characteristics and assess the risk it poses in terms of drug resistance transmission.

## 2. Materials and Methods

### 2.1. Bacterial Strains and Identification

The *P. aeruginosa* strain MAS152 (CHPC/NPRC1.9622) was isolated from the bronchoalveolar lavage fluid (BALF) of a patient in Ma’anshan, Anhui, China, in 2022. All strains used in this study were stored in the National Pathogen Resource Center (NPRC). Bacterial identification was performed using matrix-assisted laser desorption ionization time-of-flight mass spectrometry (MALDI-TOF MS).

### 2.2. Antimicrobial Susceptibility Testing

The minimum inhibitory concentrations (MICs) for 14 antimicrobial agents were determined using the BD Phoenix™ M50 with NMIC-413. The interpretation of fosfomycin results followed EUCAST guidelines [15], while other antibiotics were interpreted following the CLSI guidelines [16]. *P. aeruginosa* ATCC27853 was used as a control strain.

### 2.3. Genome Sequencing and Assembly

Genomic DNA was extracted using the Wizard Genomic DNA Extraction Kit (Promega, Madison, WI, USA) and sequenced on the Illumina NovaSeq6000 and Oxford Nanopore Technologies MinION platforms. We utilized Sickle (https://githubcom/najoshi/sickle, accessed on 2 April 2023) for raw data processing and Unicycler (https://github.com/rrwick/Unicycler, accessed on 5 May 2023) for hybrid assembly after quality control [17].

### 2.4. Bioinformatics Analysis

Whole-genome sequencing (WGS) data were analyzed using various databases and tools. EzBioCloud databases (https://www.ezbiocloud.net/tools/ani, accessed on 12 June 2023) confirmed bacterial strain identities through Average Nucleotide Identity (ANI) calculation. MLST2.0 databases were used to perform multilocus sequence typing (MLST), and the Resfinder4.1 databases (https://cge.food.dtu.dk/services/ResFinder/, accessed on 12 June 2023) were used to search for resistance genes. Prokka1.14.6 and RAST2.0 (http://rast.nmpdr.org/, accessed on 13 June 2023) combined with Pfam and NCBI-NR databases were used for annotation. Type II toxin–antitoxin (TA) systems were predicted using TAfinder (https://bioinfo-mml.sjtu.edu.cn/TAfinder/index.php, accessed on 14 June 2023). BLAST in the NCBI database aided in identifying sequences with high plasmid similarity. BRIG and Easyfig were used for sequence comparisons and map generation [18].

### 2.5. Plasmid Conjugation Assay

The conjugation assay was performed by using MAS152 as the donor and rifampicin-resistant *P. aeruginosa* PAO1 as the recipient, to identify the self-transfer ability of the plasmid pMAS152. Rifampicin (32 μg/mL) and aztreonam (8 μg/mL) were used for selection. Successful conjugation was confirmed by susceptibility testing, ARG detection, and MLST of suspected transconjugants. The calculation formula for conjugation frequency is expressed as follows: conjugation frequency = (number of transconjugant bacterial colonies × 10^x^/number of donor bacterial colonies × 10^y^), where ‘x’ and ‘y’ represent the dilution factors. The growth kinetics of stains were detected and recorded using the Bioscreen fully automated microbial growth curve analyzer for growth conditions of 37 °C and measured at OD_600_.

### 2.6. Phylogenetic Analysis of pMAS152-like Plasmids

Plasmid sequences with at least 80% homology to the plasmid pMAS152 sequence in NCBI (*n* = 60) were screened for phylogenetic tree construction (Table S1). GGDC 3.0 (Genome to Genome Distance Calculator 3.0, https://ggdc.dsmz.de/ggdc.php#, accessed on 6 November 2023) was employed to generate distance matrices, using the online website FastMe2.0 (http://www.atgc-montpellier.fr/fastme/, accessed on 8 November 2023) based on NJ method to infer phylogeny, and using chiplot for beautification [19].

### 2.7. Nucleotide Sequence Accession Number

The complete sequences of pMAS152 and the chromosomes of MAS152 were submitted to GenBank under accession numbers CP139037 and CP139036, respectively. Additionally, the complete genome sequence of strain MAS15286 is available at the National Microbiology Data Center (www.nmdc.cn, accessed on 16 November 2023) with the number NMDC60139507.

## 3. Results

### 3.1. Antimicrobial Susceptibility Testing of MAS152

In vitro susceptibility testing revealed that *P. aeruginosa* MAS152 was only sensitive to colistin, a last-line antibiotic, and had resistance to 13 agents across 7 antibiotics (Table 1). These include aminoglycosides, antipseudomonal carbapenems, cephalosporins, fluoroquinolones, penicillins + β-Lactamase inhibitors, monobactams, and phosphonic acids. Based on the non-susceptibility to at least six categories, MAS152 was classified as an XDR strain [9].

### 3.2. Genomic Features of MAS152 and Plasmid pMAS152

**Figure 1:**
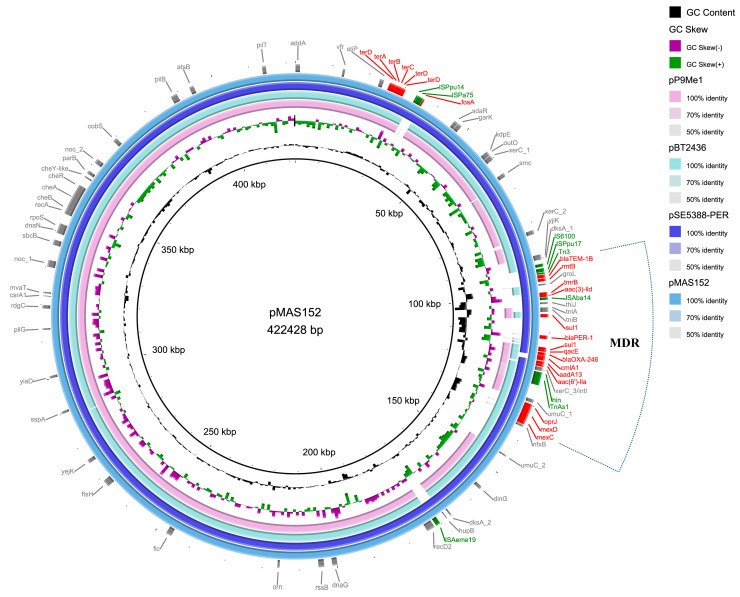
Comparative structural analysis of pMAS152 with other similar plasmids available in the NCBI nr database. The outermost circle represents the reference plasmid pMAS152. Genes involved in antimicrobial and heavy metal resistance are red, mobile elements are green, and genes involved in other functions are gray.

The genome of MAS152 consists of a 7,026,323 bp circular chromosome with a GC content of 65.89% and is identified as MLST sequence type ST357. The plasmid, pMAS152, 422,428 bp with 56.63% GC content, has 538 predicted coding sequences, of which 72% encode proteins of undetermined function (Figure 1 and Table S2). Comparing the complete sequence of pMAS152 with the plasmid sequence in Genbank, pMAS152 has the highest similarity to the plasmid pSE5388-PER (accession number: MT598646.1) from *P. aeruginosa* stain SE5388 (both identity and coverage can reach 99%). In addition, pMAS152 has high similarity with the plasmids pTJPa150 (CP094678.1), pBT2436 (CP039989.1), and pP9Me1 (CP118639.1), all carried by *P. aeruginosa* and isolated from China, Thailand, and Egypt (Figure 1). The main difference between the plasmid pMAS152 and its similar plasmids lies in the region where the resistance genes gather, as well as the region where conjugation-related genes are distributed.

Plasmid pMAS152 is classified under the incompatibility group IncpP-2A and contains a sole RIP (replication initiation protein) gene (repP-2A) with a nucleotide sequence exhibiting 100% identity to the RIP gene found in the plasmid Rms139 (accession number: LC653116.1). In addition, the plasmid pMAS152 backbone contains partitioning (*parA*, *parB,* and *parM*) and transfer (*traB*, *traV*, and *virB4*) genes. Importantly, there is a pair of type II TA systems on the plasmid pMAS152, SlvT/SlvA, which is 100% homologous to SlvT/SlvA on the plasmid pTTS12 (accession number: CP009975.1) carried by *P. putida* S12, which can maintain plasmid stability. Furthermore, the plasmid pMAS152 carries twitching motility genes (*chpA*, *pilJ*, *pilB,* and *pilT*), chemotaxis genes (*cheB*, *cheR,* and *cheY*), and heavy metal tellurium resistance genes (*terABCD*).

Besides the backbone region, the plasmid pMAS152 carries thirteen kinds of ARGs, including four aminoglycoside resistance genes (*aac(3)-IId*, *rmtB*, *aac(6′)-IIa*, *aadA1*), three β-Lactamase resistance genes (*bla*_PER-1_, *bla*_TEM-1B_, *bla*_OXA-246_), one sulfonamide resistance gene (*sul1*), one quaternary ammonium compound resistance gene (*qacE*), one chloramphenicol resistance gene (*catB*), one tunicamycin resistance gene (*tmrB*), one gene (*cmlA8*) encoding MFS transporter, and one operon *tmexCD-oprJ* encoding the antibiotic efflux pump. The MFS transporter from *cmlA8* can mediate chloramphenicol resistance, while the antibiotic efflux pump from *tmexCD-oprJ* can mediate resistance to multiple antibiotics, especially the fluoroquinolone antibiotics.

### 3.3. Multidrug Resistance (MDR) Region of pMAS152

**Figure 2:**
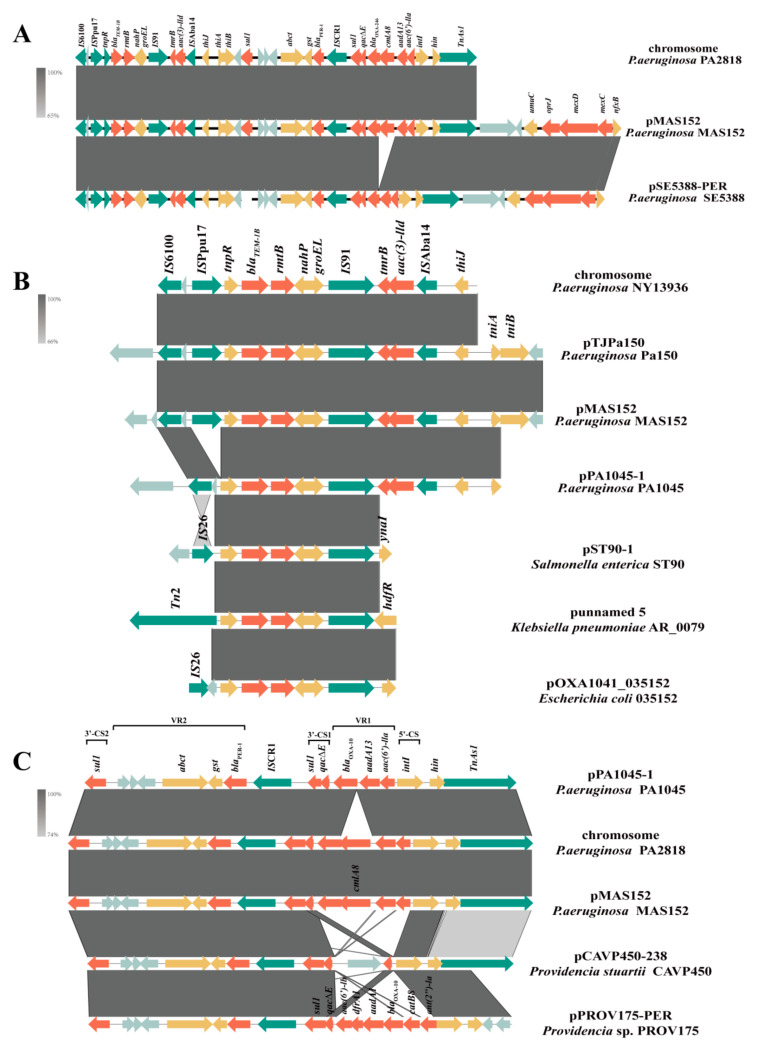
Schematic presentation of MDR region. Genes are presented as arrows, with the arrowhead indicating the direction of transcription. Genes involved in antimicrobial resistance are in orange, mobile elements are in green, genes involved in other functions are in yellow, and genes involved in undetermined coding functions are in light gray. Homologous segments generated by a BLASTn comparison (>65% identity) are gray boxes. Alignment of genetic contexts of the MDR region with a length of 44.8 kb (**A**), *rmtB* (**B**), and *bla*_PER-1_ (**C**) in the plasmid pMAS152-MDR with similar sequences.

The MDR region of pMAS152 spans 44.8 kb with 40 CDs and a 56% GC content. All ARGs carried on the plasmid pMAS152 are located in this region, and multiple mobile elements are present on both sides and in the middle of this sequence, including *IS*6100, *IS*Ppu17, *IS*Aba14, *Tn*3, and *Tn*As1. This can indicate that ARGs in this region were obtained through horizontal transfer, and active acquisition events may have occurred in this region (Figure 2A). The MDR region closely resembles that of pSE5388-PER, with the primary distinction being an additional chloramphenicol resistance gene *cmlAl* in pMAS152. Additionally, the similarity between this MDR region and a segment on the chromosome of *P. aeruginosa* PA2818 (accession number: CP129688.1) is also high, with the only notable differences observed in the operon *oprJ-mexCD* encoding the efflux pump and its regulatory factor *nfxB*.

Importantly, a gene *rmtB* encoding 16S rRNA methyltransferase (16S-RMTases) can be found in the MDR region (Figure 2B). The gene structure for *tnpR-bla*_TEM-1B_*-rmtB-nahP-IS91* is always conserved, whether on the chromosomes (like the chromosome of strain NY13936 with accession number CP096964.1) or plasmids (like the plasmid pTJPa150 with accession number CP094678.1 and the plasmid pPA1045-1 with accession number CP129995.1) of *P. aeruginosa*, as well as the plasmids of other Gram-negative bacilli, like the plasmid pST90-1 of *Salmonella enterica* ST90 (accession number: CP050735.1), the plasmid of punnamed5 of *Klebsiella pneumoniae* AR_0079 (accession number: CP028996.1) and the plasmid pOXA1041_035152 of *Escherichia coli* (accession number: CP101706.1). Among them, *tnpR* and *bla*_TEM-1B_ are transposon passenger genes of *ΔTn*2. This indicates that *ΔTn*2 and *IS*91 may play an important role in the dissemination of the gene *rmtB*. On the contrary, the mobile genetic elements (MGEs) upstream of the gene *rmtB* are diverse in different strains, like *IS*Ppu17, *IS*26, *Tn*2, and *IS*26, indicating that this conserved structure can bind to a variety of MGEs and actively spreads between strains.

Additionally, a *bla*_PER-1_ gene encoding extended-spectrum β-Lactamase (ESBL) was also found in the MDR region, which was located upstream of the *Tn*3 family transposase *Tn*As1 and in a complex Class I integron, with the gene arrangement *intI-aac(6′)-IIa-aadA13-bla*_OXA-10_*-qacE∆1-sul1-ISCR1-bla*_PER-1_*-gst-abct-sul1* (Figure 2C). Through BLASTn, this complex Class I integron can be found on both the chromosome (chromosome of strain PA2818 with accession number CP129688.1) and plasmid (pPA1045-1 with accession number CP129995.1) of *P. aeruginosa*, with high similarity (>93% identity at 99%). Moreover, the consistent position of this Class I integron upstream of *Tn*As1 suggests a potential role for *Tn*As1 in facilitating the horizontal transmission of *bla*_PER-1_ within *P. aeruginosa* through the transfer of the complex Class I integron. The left and right sides of IS*CR1* exhibit completely different conservatism. The structure on its left, *bla*_PER-1_-*gst-abct*, is relatively conserved in plasmids of both *P. aeruginosa* and other Gram-negative bacilli, like *Providencia* sp. (pCAVP450-238 of strain CAVP450 with accession number CP119553.1 and pPROV175-PER of stain PROV175 with accession number CP120534.1), while the resistance genes carried on the right side of IS*CR1* show significant differences.

### 3.4. Conjugation Transfer Experiment

The conjugation experiment confirmed the transfer of pMAS152 to *P. aeruginosa* PAO1, with a frequency of 3.3 × 10^−7^ transconjugants per donor cell. The transconjugant T152 exhibited increased resistance to various antibiotics (Table 1) and harbored *bla*_PER-1_, *bla*_TEM-1B_, *aac(3)-IId*, *aac(6′)-IIa,* and *rmtB.* Notably, these genes were absent in the recipient strain PAO1. Both PAO1 and T152 were identified as MLST type ST549, while MAS152 is ST357. The above three points help confirm the successful conjugation of the plasmid pMAS152 to the recipient strain PAO1.

**Figure 3:**
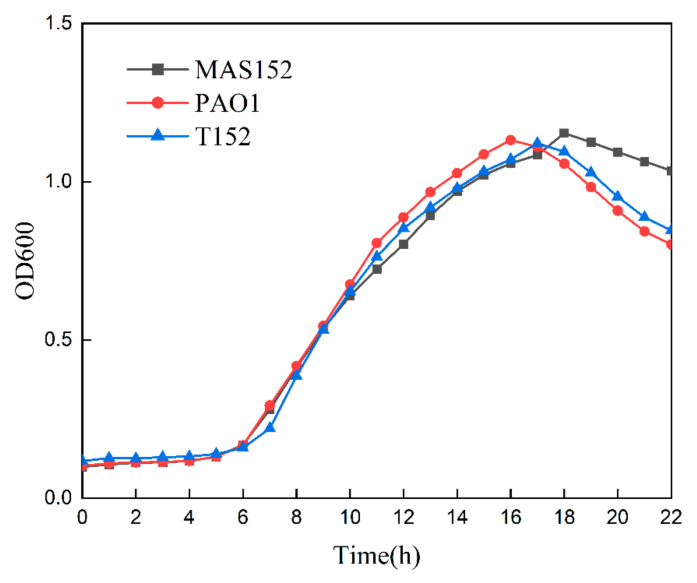
Growth curve of the donor stain MAS152, the recipient stain PAO1, and the transconjugant T152.

Growth kinetics studies have shown that there is no significant difference between the donor strain MAS152, recipient strain PAO1, and transconjugant T152 by comparing the lag phase and logarithmic phase across strains (Figure 3). In contrast to the donor strain MAS152, which exhibits a prolonged and gradual stationary phase, both the receptor strain PAO1 and the transconjugant T152 demonstrate a minimal stationary phase and rapidly transition into the decline phase following the logarithmic phase. The highly similar growth curves of the receptor strain PAO1 before and after conjugation indicate that the plasmid may not impose an additional growth burden on the receptor bacteria.

### 3.5. Phylogenetic Analysis of pMAS152-like Plasmids

**Figure 4:**
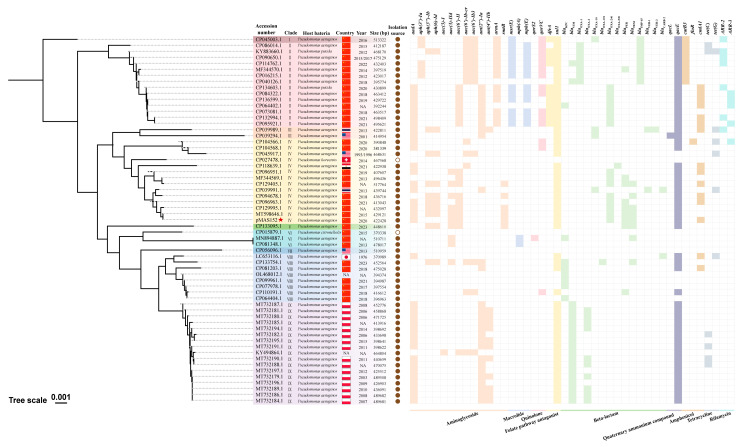
Phylogenetic tree and distribution of antibiotic resistance genes of pMAS152-like plasmids. Brown solid circles represent plasmids that were isolated from human samples, while brown hollow circles represent those isolated from non-human samples. In the heatmap of ARGs, if there is a color block, it indicates the presence of an ARG, and if there is a blank, it indicates the absence of an ARG. The red star indicated that this was the XDR strain MAS152 identified in this study.

Phylogenetic analysis showed that these 61 plasmids can be clustered into nine clades. All of them were isolated from the genus *Pseudomonas* (Figure 4), with *P. aeruginosa* as the main host strain (57, 93.4%). Furthermore, although these plasmids have been isolated globally, a significant portion (35, 57.4%) of them originated from China. Meanwhile, among these host strains, except for one plant sample (accession number: CP027478.1) from *Origanum marjorana* and one environmental sample (accession number: CP015879.1) from sludge, the rest were isolated from different samples of patients. This indicates that pMAS152-like plasmids are capable of breaking through the barriers of different species within the genus of *Pseudomonas* for cross-host and cross-regional transmission, and may be able to transmit and widely spread between the environment and humans, with potential specific transmission patterns and host preferences. However, it is important to acknowledge that this observation may be influenced by varying detection intensities, and biases cannot be ruled out entirely.

The analysis of ARGs within pMAS152-like plasmids revealed substantial variability in the ARGs carried by them, and a discernable correlation exists between the branches of the phylogenetic tree and the distribution of ARGs. For example, plasmids within clade Ⅱ exhibit greater diversity and abundance of ARGs. Among them, genes that mediate quinolone and macrolide antibiotic resistance are mostly located in this branch. In clade Ⅸ, plasmids isolated from Poland all carry *bla*_VIM_ but do not carry amphenicol-mediated resistance genes, which is a significant difference compared to plasmids from other branches. Importantly, among the plasmids closely related to pMAS152, there is a striking similarity in ARG profiles and all these plasmids carry key genes including *rmtB*, *bla*_PER_, and *bla*_OXA-246_.

## 4. Discussion

Plasmids are classified by their incompatibility groups, which indicate their inability to coexist stably in the same host cell due to similar replication and partition systems. Inc groups IncP-1 to IncP-14 are known to have all been located within *Pseudomonas* plasmids [20]. In recent years, the IncP-2A plasmids frequently found in antibiotic-resistant *Pseudomonas* often carry significant AMR genes [21,22]. Our study focuses on plasmid pMAS152, an IncP-2A plasmid, which harbors a multitude of ARGs including *rmtB, bla*_PER-1,_ and t*mexCD-opr*J, alluding to its role in multidrug resistance.

*P. aeruginosa* can mediate resistance to aminoglycoside antibiotics through efflux pump action, biofilm formation, production of aminoglycoside-modifying enzymes (AMEs), and production of 16S-RMTases [23]. Among these characteristics that help *P. aeruginosa* combat resistance, the most clinically significant resistance mechanism is the acquisition of 16S-RMTases, as they can mediate high levels of resistance to clinically relevant aminoglycoside drugs using only a single gene [24,25]. RMTB is one of the 16S-RMTases which can develop resistance to 4,6-disubstituted 2-deoxylamine (DOS) aminoglycoside drugs, including kanamycin, gentamicin, tobramycin, and amikacin, but cannot induce resistance to 4,5-disubstituted 2-DOS agents, such as neomycin and paromycin [26]. In the UK, *rmtB* is the gene with the highest detection rate among the 16S-RMTases mentioned above in *P. aeruginosa* [27]. In China, *rmtB* is also the main prevalent genotype of 16S-RMTase and is often present in combination with *armA* [28]. Further genetic environment studies of *rmtB* in pMAS152 show that *rmtB* is located downstream of *ΔTn*2 (*tnpR-bla*_TEM-1B_), which is consistent with the classical genetic environment of *rmtB* [29]. In addition, we also found that the genetic environment *nahP-groEL-IS*91, located downstream of *rmtB*, is relatively conserved on plasmids in both *P. aeruginosa* and other Gram-negative bacilli, presenting the conserved genetic environment *ΔTn*2-*rmtB*-*nahP-groEL-IS*91. This phenomenon suggests that in addition to *ΔTn*2, *IS*91 may also play an important role in the spread of the gene *rmtB*. Compared to this conservative genetic environment, the genes located downstream of *IS*91 have significant differences between plasmids, mostly consisting of various ARGs or functional genes. Variations downstream of *IS*91 suggest active transfer and transmission of *rmtB* among different strains. Moreover, upstream of *ΔTn*2, different mobile elements such as *IS*6100, *IS*26, and *Tn*2 can often be found, which, in combination, can lead to easier transfer and transmission of *rmtB* between different strains [30].

The production, evolution, and spread of β-Lactamases are the main mechanisms by which *P. aeruginosa* is resistant to β-Lactam drugs [31]. According to the Bush–Jacoby–Medeiros functional system, β-Lactamases can be divided into five types [32]. Within these systems, ESBLs are predominately plasmid-mediated enzymes, capable of hydrolyzing antibiotics such as penicillin, cephalosporins (mainly third-generation cephalosporins such as ceftazidime, cefoperazone, etc.), and monobactams. However, their hydrolysis ability for carbapenems and cephalosporins (e.g., cefoxitin and cefmetazole) is comparatively weak. PER-1, as one of the ESBLs, was largely detected across nonfermentative Gram-negative bacilli, with it being detected the most in *Acinetobacter baumannii*, followed by *P. aeruginosa* and *Providencia alcalifaciens*. Currently, *bla*_PER-1_ is widely distributed in strain isolates across Asia and Europe [33,34]. In this study, the results of the conjugation assay revealed that upon acquiring the plasmid pMAS152, the transconjugant T152 exhibited resistance to all β-Lactam antibiotics except carbapenems, which is consistent with the resistance profile mediated by *bla*_PER-1_. The insensitivity of the strain MAS152 to carbapenems may be related to resistance mechanisms on chromosomes, such as the absence or mutation of the outer membrane porin OprD and the action of efflux pump MexAB-OprM [35]. In the past, the genetic environment of *bla*_PER-1_ was believed to be related to IS*Pa12* and IS*Pa13*, which collectively form the composite transposon Tn*1213*, while the promoter P_IS*Pa12*_ in IS*Pa12* can drive the expression of *bla*_PER-1_ [36]. In recent years, a complex Class I integron containing IS*CR1* has been reported to play a powerful genetic vehicle role in the dissemination of *bla*_PER-1_, and IS*CR1*- *bla*_PER-1_-3′-CS may actually mediate the formation of this complex Class I integron [37]. In addition, the transcription level of *bla*_PER-1_ is also affected by IS*Pa1635*, which is inserted between IS*CR1* and *bla*_PER-1_. Insertion of IS*Pa1635* may lead to increased resistance potential of *P. aeruginosa* to novel β-Lactam/β-Lactamase inhibitor combinations and cefiderocol [38]. In the plasmid pMAS152, *bla*_PER-1_ is located in a complex Class I integron containing IS*CR1*, and there is no insertion sequence between IS*CR1* and *bla*_PER-1_. This genetic environment is similar to that of *P. aeruginosa* RJ246 and RJ252, previous isolates originated from China [37]. Furthermore, we also found that in this complex Class I integron, VR2 is relatively conserved in *P. aeruginosa* and other Gram-negative bacilli, while VR1 often contains different resistance genes, which may be partially attributed to the loss and acquisition of the IS*CR1*- *bla*_PER-1_-3’-CS structure.

So far, the *rmtB* gene has been detected in bacteria from over 30 countries worldwide [39]. Multiple studies have shown that RMTB is strongly correlated with CTX-M-type ESBLs and VIM-type carbapenemases, which are often found on the same plasmid [40,41]. Their emergence complicates the management of infections caused by multidrug-resistant strains. Our study reported a plasmid harboring both RMTB- and PER-type ESBL-encoding genes simultaneously. Additionally, the plasmid pMAS152 also carries an operon *tmexCD-oprJ* encoding an efflux pump, which mainly mediates resistance to fluoroquinolone antibiotics such as levofloxacin and ciprofloxacin [42]. No such combination has been found in the currently reported plasmids, and the combination of *rmtB*, *bla*_PER-1,_ and *tmexCD-oprJ* can make the stain resistant to almost all clinical drugs, seriously threatening the effectiveness of clinical treatment. Moreover, conjugation transfer experiments, annotations for transfer genes, and phylogenetic tree analysis revealed that pMAS152 can be characterized as a conjugative plasmid. In addition, not only does *P. aeruginosa* serve as its host bacteria, but other *Pseudomonas* species also present as potential hosts, which can further exacerbate the spread of antibiotic resistance.

The growing threat posed by ARG-positive plasmids necessitates novel strategies for plasmid curing, such as nanoparticles, engineered phages, and CRISPR/Cas technology [43,44]. These strategies provide promising assistance for the control of ARG-positive plasmids but still face many challenges in their application. Most current research experiments are conducted on the basis of a single type of cell in vitro, and there is a lack of experimental data based on population structure in vivo. In addition, these strategies require detailed characterization of functional regions of the plasmid, especially the replication, separation, and conjugation regions. More efforts are needed to translate these strategies into clinical practice.

## 5. Conclusions

We characterized an XDR clinical *P. aeruginosa* strain MAS152 (CHPC/NPRC1.9622) carrying multiple ARGs, including *rmtB*, *bla*_PER-1,_ and *tmexCD-oprJ*. These ARGs are all located on the unique plasmid pMAS152 of the strain MAS152. It seems to be the first plasmid reported that simultaneously carries these three important ARGs, and obtaining such a plasmid could render common clinically used first-line antibiotics ineffective against *P. aeruginosa* infections. Our research emphasized that different ARGs can be combined on plasmids and then spread to different host bacteria with plasmids as vehicles, resulting in an increasing number of antibiotic-resistant bacteria. Besides that, our research enriches the library of antibiotic-resistant plasmids and provides a foundation for drug resistance monitoring and subsequent plasmid-curing research. In the present and future, measures like strengthening experimental research based on the population structure in vivo*,* improving antibiotic management, and expanding the scope of surveillance should be taken to prevent the spread of such plasmids.

## Figures and Tables

**Table 1:** Antimicrobial resistance pattern of *Pseudomonas aeruginosa* strains MAS152, PAO1 and T152.

Antimicrobial Class/Agent	MIC (µg/mL) [Susceptibility]		
	MAS152	PAO1	T152
Aminoglycosides			
Amikacin	>32[R]	≤8[S]	>32[R]
Gentamicin	>8[R]	≤2[S]	>8[R]
Tobramycin	>8[R]	≤2[S]	>8[R]
Antipseudomonal carbapenems			
Imipenem	>8[R]	4[I]	8[R]
Meropenem	>8[R]	1[S]	2[S]
Antipseudomonal cephalosporins			
Cefepime	>16[R]	≤1[S]	>16[R]
Ceftazidime	>32[R]	2[S]	>32[R]
Antipseudomonal fluoroquinolones			
Ciprofloxacin	4[R]	2[R]	4[R]
Levofloxacin	8[R]	4[R]	8[R]
Norfloxacins	>8[R]	8[I]	>8[R]
Antipseudomonal penicillins + β-Lactamase inhibitors			
Piperacillin–Tazobactam	>64/4[R]	≤4/4[S]	>64/4[R]
Monobactams			
Aztreonam	>32[R]	≤2[S]	>32[R]
Phosphonic acids			
Fosfomycin w/G6P	>128[>ECOFF]	128[≤ECOFF]	128[≤ECOFF]
Polymyxins			
Colistin	≤1[S]	≤1[S]	≤1[S]

Note: R, resistant; I, intermediate; S, susceptible; ECOFF, epidemiological break point based on in vitro antimicrobial sensitivity data.

## Data Availability

The complete sequences of pMAS152 and the chromosomes of MAS152 were submitted to GenBank under accession numbers CP139037 and CP139036, respectively. Additionally, the complete genome sequence of strain MAS15286 is available at the National Microbiology Data Center (www.nmdc.cn) with the number NMDC60139507.

## Supplementary Materials

The following supporting information can be downloaded at https://www.mdpi.com/article/10.3390/microorganisms12030562/s1: Table S1: Basic information of pMAS152-like plasmids; Table S2: Annotation information of plasmid pMAS152.
